# Pyramiding Breeding of Low-Glutelin-Content *Indica* Rice with Good Quality and Resistance

**DOI:** 10.3390/plants12213763

**Published:** 2023-11-03

**Authors:** Da-Gang Chen, Jie Guo, Ke Chen, Chan-Juan Ye, Juan Liu, You-Ding Chen, Xin-Qiao Zhou, Chuan-Guang Liu

**Affiliations:** 1 Rice Research Institute, Guangdong Academy of Agricultural Sciences, Guangzhou 510640, China; chendg@gdaas.cn (D.-G.C.); guojie@gdaas.cn (J.G.); chenke@gdaas.cn (K.C.); yechanjuan@gdaas.cn (C.-J.Y.); liujuan@gdaas.cn (J.L.); chenyd@gdaas.cn (Y.-D.C.); 2 Key Laboratory of Genetics and Breeding of High Quality Rice in Southern China (Co-Construction by Ministry and Province), Guangzhou 510640, China; 3 Ministry of Agriculture and Rural Affairs, Guangdong Key Laboratory of New Technology in Rice Breeding, Guangzhou 510640, China; 4 Guangdong Rice Engineering Laboratory, Guangzhou 510640, China

**Keywords:** low-glutelin-content, resistance, good quality, *indica* rice, pyramiding breeding

## Abstract

Low-glutelin-content rice, a type of functional rice with glutelin levels below 4%, is an essential dietary supplement for chronic kidney disease (CKD) patients. Developing low-glutelin-content rice varieties is crucial to catering to the growing CKD population. In this study, we aimed to create a new low-glutelin *indica* rice variety with excellent agronomic traits. To achieve this, we employed a combination of molecular-marker-assisted selection and traditional breeding techniques. The cultivars W3660, Wushansimiao (WSSM), and Nantaixiangzhan (NTXZ) were crossbred, incorporating the *Lgc-1*, *Pi-2*, *Xa23*, and *fgr* alleles into a single line. The result of this breeding effort was “Yishenxiangsimiao”, a new *indica* rice variety that inherits the desirable characteristics of its parent lines. Yishenxiangsimiao (YSXSM) possesses not only a low glutelin content but also dual resistance to blast and bacterial blight (BB). It exhibits high-quality grains with a fragrant aroma. This new low-glutelin *indica* cultivar not only ensures a stable food supply for CKD patients but also serves as a healthy dietary option for the general public. We also performed RNA-seq of these rice varieties to investigate their internal gene expression differences. The YSXSM exhibited a higher biotic-resistance gene expression in comparison to NTXZ. In summary, we successfully developed a novel low-glutelin *indica* rice variety, “Yishenxiangsimiao”, with superior agronomic traits. This rice variety addresses the dietary needs of CKD patients and offers a nutritious choice for all consumers.

## 1. Introduction

Diabetes mellitus has emerged as one of the most serious and prevalent chronic diseases in the 21st century, leading to life-threatening complications, disabilities, and a reduction in life expectancy [1,2,3]. Moreover, it has evolved into a global health crisis. The International Diabetes Federation (IDF) reported that in 2021, an estimated 536.6 million people were living with diabetes, and this number is projected to surge by 46% to reach a staggering 783.2 million by 2045 [4]. This alarming increase is primarily attributed to rapid urbanization, shifts in dietary patterns, and a rise in sedentary lifestyles [5]. What is even more concerning is that nearly half of all adults with diabetes are unaware of their diabetic status [6]. Chronic kidney disease (CKD) is among the numerous chronic conditions triggered by diabetes mellitus [7,8], and it has become a pervasive issue worldwide, particularly in developing nations [9,10]. According to a recent survey, China alone has approximately 119.5 million cases of chronic kidney disease, with a prevalence rate of 10.8% [11]. Given this alarming prevalence, it is imperative that the scientific community directs significant attention to this chronic disease and its associated complications.

Rice (*Oryza sativa* L.) stands as one of the world’s foremost staple cereals, providing sustenance to more than half of the global population, with over 60% of China’s population relying on it for sustenance. Furthermore, it contributes to nearly 40% of the nation’s total calorie intake [12,13]. Among the essential nutritional components in rice grains, protein plays a pivotal role. The protein content in rice grains typically ranges from 8% to 10%, encompassing various types such as glutelin, gliadin, albumin, and globulin. Of these, glutelin is the most prevalent digestible component of rice storage protein, constituting a substantial 80% of the total protein content in rice grains [14]. However, excessive consumption of glutelin-rich rice can exacerbate medical issues for CKD patients. The majority of these patients are advised to avoid rice with more than 4% soluble protein content [15]. Consequently, individuals who rely on rice as their dietary staple, particularly CKD patients, are confronted with a dilemma. They must either continue consuming rice and potentially compromise their health or make significant alterations to their dietary habits. To address this predicament, the development of rice varieties with a low glutelin content, suitable for the daily consumption of a large CKD population, becomes of paramount importance.

The first low-glutelin-content rice variety, “LGC1”, was created through mutation breeding by the National Institute of Agrobiological Sciences (NIAS) from the “Nihonmasari” rice variety [16]. LGC1 exhibited a significant decrease in the glutelin content with a simultaneous increase in the prolamine content compared with the wild type. Research indicated that the trait of a low glutelin content was controlled by a single dominant gene, with the mutation locus *Lgc1*, situated between the markers XNpb243 and G365 on chromosome 2 [17]. This low-glutelin effect was attributed to RNA interference and a 3.5 kb deletion between the *GluB4* and *GluB5* genes, resulting in a tail-to-tail inverted molecule formation. This molecular alteration triggered post-transcriptional gene silencing, leading to the observed consequences [18]. Subsequently, scientists developed a mutant line that was deficient in 26 KDa globulin from “Koshihikari.” Two new varieties, “LGC-Katsu” and “LGC-Jun”, were created through a cross between LGC-1 and the mutant [19]. “W3660”, another low-glutelin rice variety, was developed through a cross between LGC-1 and “Koshihikari” using marker-assisted and recurrent selection [20]. Further genetic improvement was aimed at enhancing W3660’s yield and resistance, resulting in the emergence of “W0868”, a new variety with improved yield and modified agronomic characteristics, which was eventually released for commercial applications. Research demonstrated that this rice variety significantly reduced postprandial blood glucose and urinary protein levels in rodents, thus protecting renal function without compromising nutritional status [21,22]. Through fine-mapping and gene cloning related to glutelin in rice [18,23,24,25,26], the genetic and molecular regulation mechanisms of low glutelin have been elucidated. Several functional molecular markers have been designed for the assisted selection of low-glutelin rice based on these studies [24,27,28,29]. Moreover, numerous new low-glutelin rice varieties have been developed in recent years through marker-assisted selection (MAS) [29,30,31,32] or CRISPR/Cas9 gene editing technology [33,34]. However, it is worth noting that most of the low-glutelin-content rice varieties are currently of the *japonica* species, with very few reports on the breeding of low-glutelin *indica* rice.

In certain regions, such as South China, there is a growing demand for low-glutelin *indica* rice, particularly from diabetes patients. In previous research, an indica rice variety with a low glutelin content derived from “W3660” was developed through MAS [35]. However, it was observed that the appearance and cooking quality of this variety were suboptimal. To address this, a new *indica* rice variety, “Yishenxiangsimiao” (YSXSM), was created by crossing the low-glutelin *japonica* “W3660” with the normal-glutelin *indica* “Wushansimiao” (WSSM), which also had blast and bacterial blight resistance. Further improvement of rice quality was achieved through hybridization with “Nantaixiangzhan” (NTXZ), known for its good appearance and fragrance. Progeny were selected using MAS and traditional breeding practices, resulting in the development of YSXSM. This new *indica* rice variety boasts a glutelin content of less than 4% and strikes a balance between yield, disease resistance, and rice quality inherited from its parent lines. Our research data indicate that YSXSM not only provides a stable food source suitable for CKD patients but also serves as a healthy dietary option for the general public.

## 2. Materials and Methods

### 2.1. Plant Materials

W3660 was crossed with WSSM, an *indica* variety developed by the Rice Research Institute of Guangdong Academy of Agricultural Sciences (GDRRI), with a normal glutelin content, high yield, and disease resistance in 2011. The F_1_ plants were screened for the heterozygosity for the *Lgc-1*, *Pi-2*, and *Xa23* alleles, and then one plant that carried the targeted alleles was selected for backcrossing with WSSM until the BC_3_ population was achieved. A line that integrated the alleles of *Lgc-1*, *Pi-2*, and *Xa23* alleles and had good agronomic traits was selected from the BC_3_F_4_ generation in 2014 and crossed with NTXZ, an elite *indica* variety developed by GDRRI with a normal glutelin content and high quality. An F_2_ population consisting of 1000 individuals was developed from the F_1_ plant that was heterozygous for the *Lgc-1*, *Pi-2*, *Xa23*, and *fgr* alleles in 2015. The homozygous alleles of the four desired genes were selected from the F_2_ population using the MAS method, and then self-pollinated up to the F_8_ inbred lines until 2018. The progeny populations, along with the parental lines, were grown in the field at GDRRI in Guangzhou with conventional water and fertilizer management. For each season of planting, we used 200 plants for marker-assisted selection for each population. The growth condition covered for two growth seasons each year, the spring season from March to July in Guangzhou, and from July to October in Guangzhou (23.158213° N, 113.371113° E). The growth location was different from that of the blast resistance trial (23.876843° N, 114.011398° E). 

### 2.2. DNA Extraction and Marker Analysis

The rice genomic DNA was extracted from the leaves of each plant among 15-day- old seedlings according to the method by Zheng et al. [36]. Sequences of the marker primers are shown in Table S1. The PCR conditions were slightly modified according to the previous method [37]. The 20 μL reaction system included 0.15 μmol/L primers, 200 μmol/L dNTP, 1× buffer (50 mmol/L KCl, 10 mmol/L Tris-HCl pH 8.3, 1.5 mmol/L MgCl_2_, 0.01% gelatin), 50–100 ng DNA template, and 1 U Taq enzyme. The reaction procedure was as follows: pre-denaturation at 94 °C for 4 min and denaturation at 94 °C for 30 s; 55 °C annealing for 30 s and 72 °C extension for 30 s, for a total of 35 cycles; 72 °C for 10 min. The reaction product was electrophoresed on 8% polyacrylamide or 2% agarose gel, stained with GelRed nucleic acid stain (Beyotime, D0139, Shanghai, China), and observed on an ultraviolet gel imager (Tanon 4600, Shanghai, China).

### 2.3. SDS-PAGE Analysis of Endosperm Protein

The total seed protein was extracted according to a previously reported method [38]. This assay used the seeds that were harvested from the field. Milled grain powder (50 mg) of each material was weighed and transferred into a 1.5 mL centrifuge tube. The SDS-PAGE method was modified from a previous method [39]. One milliliter of protein extraction buffer (50 mmol/L Tris-HCl pH 6.8, 8 mol/L urea, 4% SDS, 5% β-mercaptoethanol, and 20% glycerol) was added to each tube, adequately vortexed for several seconds (Scientific Industries, Vortex-Genie 2, Ocala, FL, USA), and shaken at 25 °C for 12 h. The supernatant was transferred into a 5 mL centrifuge tube after centrifugation at 10,000 r/min for 10 min. Ten microliters of supernatant was used for SDS-PAGE electrophoresis (separation gel 15%, concentrated gel 7.5%). The gel was soaked in fixative (10% acetic acid, 40% ethanol, 50% H_2_O) for 2 h and stained with coomassie brilliant blue R-250 (CBB-250, Bio-Rad, 161-0436, Hercules, CA, USA).

### 2.4. Protein Component Determination

The total protein content was determined using a Kjeldahl apparatus (8400, FOSS) with a conversion factor of 5.95. The extraction and determination of the components were carried out according to a previously described method [40]. Milled grain flour (0.5 g) was used, and four types of proteins were obtained according to a published method. The extraction solution components were as follows: albumin (10 mmol/L Tris-HCl, pH 7.5), globulin (1 mol/L NaCl, 10 mmol/L Tris-HCl, pH 7.5), gliadin (volume fraction 55% N-propanol, 10 mmol/L Tris-HCl, pH 7.5), and glutelin (mass fraction 0.24% CuSO_4_·5H_2_O, 1.68% KOH, 0.5% sodium potassium tartrate, and volume fraction 50% Isopropanol). Each extraction solution amounted to 25 mL and was shaken at room temperature for 2 h (Zhichu, ZHTY70, Yantai, China), followed by centrifugation at 4000× *g* for 10 min. The contents of albumin, globulin, and gliadin were detected using CBB-250, while glutelin was detected using the Biuret method [40].

### 2.5. Evaluation of Blast and Bacterial Blight

The experimental materials, including the parents and the progeny, were planted to determine the blast resistance in outdoor blast identification farmland in Guangdong Province, China (23.876843° N, 114.011398° E). The growth period and conditions were similar to those used previously, except no agricultural chemicals were applied in the field. Rice blast disease resulted from natural infection, and the cultivar Guanglu’ai was planted for rice blast induction. Bacterial blight resistance was evaluated through the leaf-clipping method [41] using the pathotype Ⅳ *Xoo* strains. All samples were cultivated in a normal field under moderate nutrient conditions in Guangzhou (23.158213° N, 113.371113° E). The statistical analysis used Student’s *t*-test to evaluate the differences in blast resistance and bacterial blight resistance among these rice varieties.

### 2.6. Evaluation of Grain Quality

Mature seeds of the experimental materials were harvested and dried, and then prepared as milled rice. Evaluation of the grain length, grain width, length/width ratio, and chalky kernels was performed according to the National Standards of the People’s Republic of China (1999) and a previous method [12]. Rice amylase, gel consistency, and crude fat were detected by the China National Rice Research Institute. The taste value was measured using an STA1B cooked rice taste meter (SATAKE, Hiroshima, Japan). The aromatic volatile 2-acetyl-1-pyrroline (2-AP) was extracted and determined with a GCMS-QP2020N (SHIMADZU, Kyoto, Japan), using 2,4,6-trimethylpyridine (TMP) as an internal standard according to the methods described by Bergman et al. [42].

### 2.7. Investigation of Agronomic Traits

All rice varieties were cultured based on the random block design principle. Three replications of plots were cultured for each rice variety. Each plot contained 200 individual plants. Plants were harvested after maturity, and agronomic traits, such as plant height, panicle length, grains per panicle, seed setting rate, 1000-grain weight, and yield per plant, were recorded and statistically analyzed using Statistical Package for the Social Sciences (SPSS) version 22.0 (IBM Corp., Armonk, NY, USA). Student’s *t*-test was used. 

### 2.8. RNA-seq and Data Analysis

RNA sequencing (RNA-seq) was performed by Biomarker Technologies Corporation (Beijing, China). We grew the four rice varieties in a climate chamber (27 °C, 13 h light; 25 °C, 11 h dark). Healthy leaves of three independent two-week-old plants were placed in liquid nitrogen and then ground and harvested for RNA library preparation. Samples of the W3660, WSSM, NTXZ, and YSXSM with three independent biological replicates were used for RNA-seq and data analysis. The raw data were processed in fastqformat using Perl scripts. We also calculated the quality score of 20 and 30 percentages (Q20 and Q30), the guanine-cytosine contents (GC contents), and sequence duplication levels from the clean data. The correlation among all samples was assessed using Pearson’s correlation coefficient [43]. For the differential expression, we employed DEseq for analysis [44]. Genes with an adjusted *p*-value < 0.01, as identified via DEseq, were considered differentially expressed genes (DEGs). Gene ontology (GO) enrichment analysis and Kyoto Encyclopedia of Genes and Genomes (KEGG) pathway enrichment analysis were performed based on Wallenius’ non-central hypergeometric distribution [45,46]. To assess the statistical enrichment of DEGs in the KEGG pathway, we used KOBAS software version 2.0 [47]. Heatmaps were generated using MeV software version 4.9.0 [48].

### 2.9. Selection Process of the New Low-Glutelin Indica Variety

**Figure 1:**
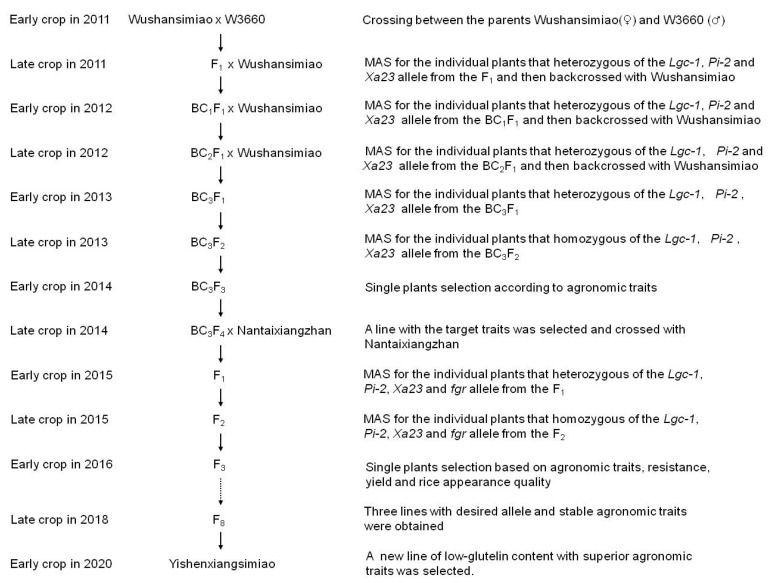
The developmental process of the new low-glutelin-content *indica* rice.

**Figure 2:**
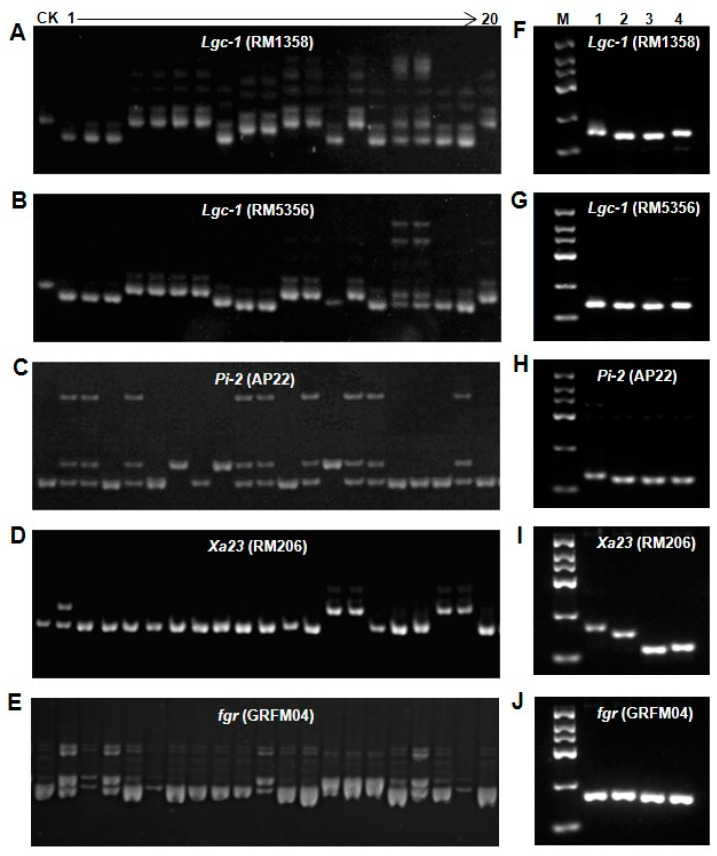
Markers-assisted selection of the *Lgc-1*, *Pi-2*, *Xa23*, and *fgr* genes. (**A**) Individual selection of *Lgc-1* (RM1358). (**B**) Individual selection of *Lgc-1* (RM5356). (**C**) Individual selection of *Pi-2* (AP22). (**D**) Individual selection of *Xa23* (RM206). (**E**) Individual selection of *fgr* (GRFM04). In (**A**,**B**), the CK is W3660; in (**C**–**E**), the CK is NTXZ. Numbers 1–20 represent partial individual plants from the F_2_ population. (**F**–**J**) show the genotypes of the new line and parents for *Lgc-1*, *Pi-2*, *Xa23*, and *fgr*. M—marker; 1 to 4 represent W3660, Wushansimiao, Nantaixiangzhan, and Yishenxiangsimiao, respectively.

The development of the excellent-quality and low-glutelin-content *indica* rice variety “Yishenxiangsimiao” (YSXSM) was guided by a comprehensive selection process (Figure 1). In 2011, 16 F_1_ hybrids were generated from the cross between “Wushansimiao” (WSSM) and “W3660”, and these were planted during the late crop season in Guangzhou. Genotyping was conducted on all 16 hybrids to identify the presence of the *Lgc-1*, *Pi-2*, and *Xa23* alleles using marker-assisted selection (MAS). Plants displaying heterozygosity for these alleles were subsequently backcrossed with WSSM to produce the BC_1_F_1_ generation. This process of backcrossing was repeated three times, ultimately leading to the creation of the BC_3_F_1_ generation by the early crop season of 2013. These individuals were then selected based on the MAS criteria, and plants displaying heterozygosity were collected to generate the BC_3_F_2_ population. From this BC_3_F_2_ population, 50 individuals were carefully chosen out of a total of 1000, considering both MAS and agronomic traits. These selected plants were self-pollinated to produce the BC_3_F_3_ and BC_3_F_4_ generations. During the generation of BC_3_F_4_, one line was identified that carried the target alleles and exhibited favorable agronomic traits. This line was then crossed with “Nantaixiangzhan” (NTXZ) during the late crop season of 2014 in Guangzhou. The F_1_ generation resulting from this cross was also genotyped for the presence of the *Lgc-1*, *Pi-2*, *Xa23*, and *fgr* alleles using MAS. The plants that displayed heterozygosity for all four alleles were self-pollinated and collected, ultimately giving rise to a total of 1000 F_2_ individuals by the late crop season of 2015. From this F_2_ population, 50 individuals were selected based on both agronomic traits and the presence of the desired alleles (*Lgc-1*, *Pi-2*, *Xa23*, and *fgr*). These individuals were then subjected to further rounds of selection, continuing the process of allele stacking and trait improvement over several generations (Figure 1 and Figure 2A–E). The SSR markers RM1358 and RM5356 [24] were used to identify the low-glutelin allele (Figure 2A,B), while the AP22 marker was employed to select for high rice blast resistance [49] (Figure 2C). The RM206 marker [50], located near the *Xa23* gene region, facilitated the selection of bacterial blight resistance (Figure 2D). Simultaneously, the gene for fragrance, *fgr*, located near the GRFM04 marker [51], was dominantly identified in the MAS-based selection (Figure 2E). Furthermore, F_3_ lines were cultivated, consisting of 40 individuals. Three samples from each F_3_ line were randomly selected for marker analysis, and the chosen individuals were self-pollinated to generate the F_4_ generation. Through continuous marker-assisted selection, homozygous lines carrying the desired alleles were obtained in the F_6_ generation, marking the late crop season of 2017. These lines were further selected based on desirable agronomic traits through traditional breeding methods. By the F_8_ generation in 2018, three individual lines emerged, each exhibiting all-homozygous genes with superior agronomic traits. To validate the success of the breeding program, various analyses were performed, including SDS-PAGE electrophoresis, protein content analysis, resistance identification, and aroma determination. Eventually, one line that contained all the desired homozygous genes with excellent agronomic traits was identified (Figure 1). In sum, we successfully bred a new *indica* rice variety that combines all the desired alleles for low glutelin content, disease resistance, and superior grain quality.

## 3. Results

### 3.1. Agronomic Traits and Yield Components of the New Line

**Figure 3:**
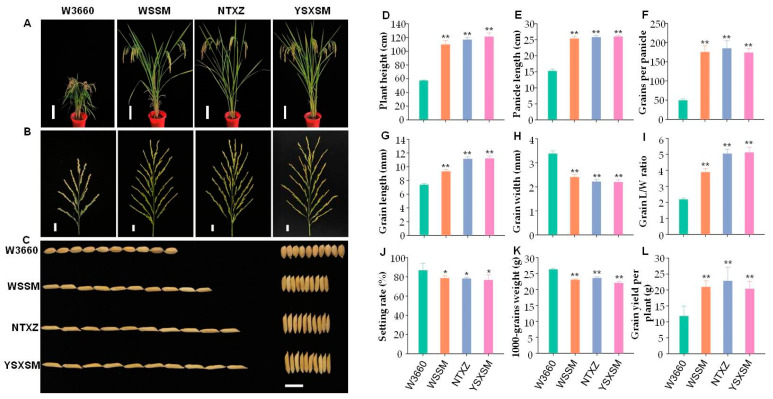
Comparison of agronomic traits of the new low-glutelin line and parents. (**A**) Plant morphology. Scale bar, 20 cm. (**B**) Main panicles. Scale bar, 2 cm. (**C**) Grain shape. Scale bar, 1 cm. (**D**) Plant height (*n* = 30). (**E**) Panicle length (*n* = 30). (**F**) Grains per panicle (*n* = 10). (**G**) Grain length (*n* = 10). (**H**) Grain width (*n* = 10). (**I**) Grain L/W ratio (*n* = 10). (**J**) Setting rate (*n* = 10). (**K**) The 1000-grain weight (*n* = 10). (**L**) Grain yield per plant. (*n* = 10). The bars indicate the standard error of the mean. * and ** represent significant difference levels of *p* < 0.05 and *p* < 0.01, respectively. WSSM—Wushansimiao. NTXZ—Nantaixiangzhan. YSXSM—Yishenxiangsimiao.

To comprehensively assess the agronomic performance of YSXSM and compare it with its parental lines, we conducted an analysis of various plant characteristics and panicle and grain morphology. The results shed light on YSXSM’s suitability for cultivation and its potential for double-cropping in South China. Firstly, when examining plant type characteristics, YSXSM exhibited similarities to the elite *indica* varieties NTXZ and WSSM. This similarity suggests that YSXSM meets the criteria for the ideal plant type of double-cropping *indica* rice in South China (Figure 3A). Analyzing the major agronomic traits and yield components, we observed that YSXSM’s plant height, panicle length, and number of grains per panicle did not significantly differ from those of NTXZ and WSSM (Figure 3B,D–F). However, it is worth noting that YSXSM displayed a significantly larger grain length and length/width ratio compared with W3660 (Figure 3C,G–I). While the setting rate of the four *indica* varieties was lower than that of W3660, statistical analysis revealed no significant differences (Figure 3J). Additionally, YSXSM’s 1000-grain weight was equivalent to that of NTXZ and WSSM, but notably lower than that of W3660 (Figure 3K). When considering the yield potential, YSXSM, while having a slightly lower yield potential than WSSM and NTXZ due to the small decrease in grain weight, demonstrated a significant improvement compared with the low-glutelin japonica “W3660” when cultivated in South China (Figure 3L). Furthermore, YSXSM displayed a growth period that was 15–20 days longer than that of W3660, equivalent to the growth period of WSSM and NTXZ. This extended growth period makes YSXSM suitable for double-cropping in South China, aligning with the region’s agricultural practices and climate conditions. In summary, extensive analysis of YSXSM’s agronomic performance indicates its suitability for cultivation in South China, particularly for double-cropping systems. This variety not only meets the ideal plant type criteria but also demonstrates competitive yields and improved grain quality compared with its parental lines, making it a valuable addition to rice cultivation in the region.

### 3.2. SDS-PAGE Electrophoresis and Component Analysis of Grain Protein

**Figure 4:**
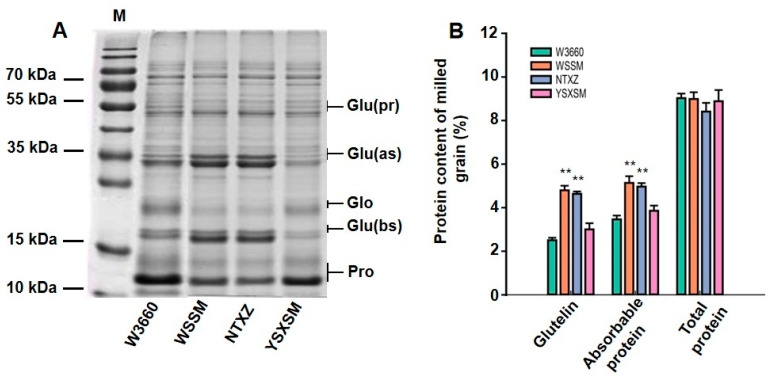
Protein component characteristics of low-glutelin lines and parents. (**A**) SDS-PAGE analysis of total protein of milled grains. M—marker; Glu(pr)—glutelin precursors; Glu(as)—glutelin acidic subunits; Glu(bs)—glutelin basic subunits; Glo—globulin; Pro—prolamin. (**B**) Determination of protein components of milled grains. The bars indicate the standard error of the mean. ** represent significant difference levels of *p* < 0.01, respectively. WSSM—Wushansimiao. NTXZ—Nantaixiangzhan. YSXSM—Yishenxiangsimiao.

In addition to assessing agronomic traits, we explored the soluble protein content of these rice cultivars to understand their nutritional profiles. The analysis included a closer look at the protein composition within the grains. When we examined the protein profile using SDS-PAGE electrophoresis, it became evident that YSXSM, inherited from W3660, had a different protein composition to NTXZ (Figure 4A). YSXSM exhibited lower levels of mature glutelin proteins (37–39 kDa and 22–23 kDa) and higher levels of prolamine (13 kDa). The quantitative analysis of the protein components further validated these differences. The glutelin content and total absorbable protein content in YSXSM and W3660 were significantly lower than those in WSSM and NTXZ, respectively. However, the overall total protein content showed similar trends among the experimental cultivars (Figure 4B). The critical point to note is that the absorbable protein content in YSXSM was below 4%, indicating that this new rice variety met the criterion for low glutelin content. These findings confirm that YSXSM is indeed a low-glutelin rice variety in terms of its grain composition.

### 3.3. Rice Blast and Bacterial Blight Resistance Evaluation

**Figure 5:**
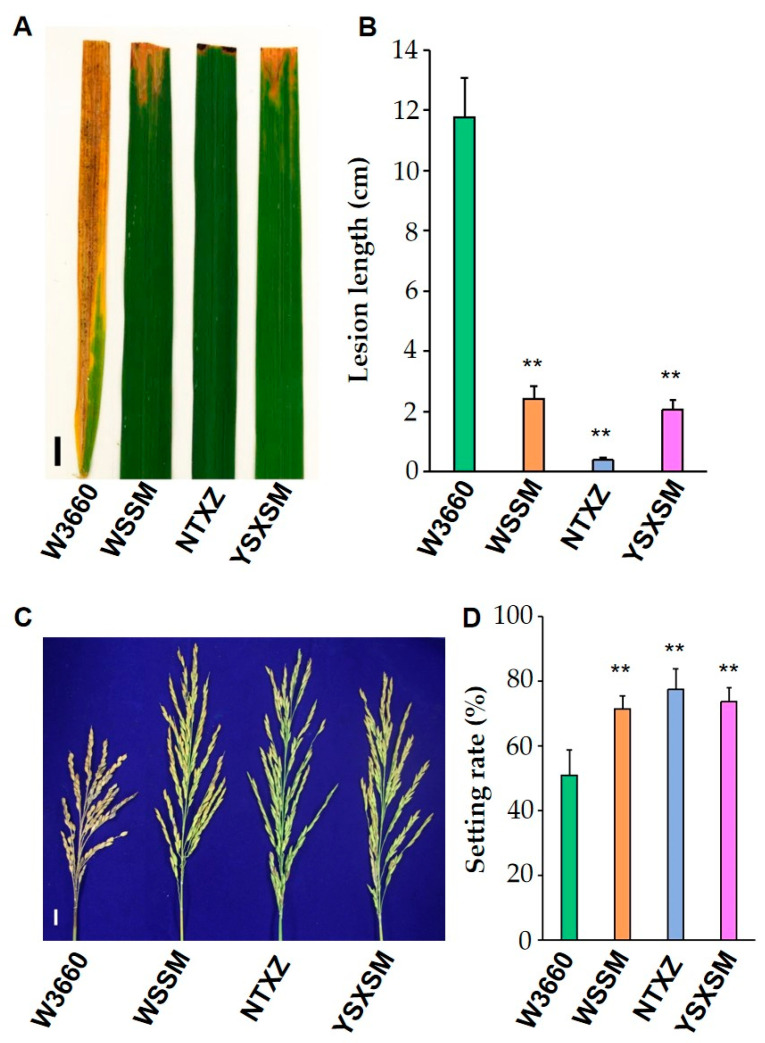
Bacterial blight and blast disease resistance evaluation of the rice varieties. (**A**) Leaf lesion patterns from the *pathotype IV Xoo* strains for W3660, WSSM, NTXZ, and YSXSM, respectively. Scale bar, 1 cm. (**B**) Statistical analysis of the leaf lesion lengths after inoculation with the *pathotype IV Xoo* strains. The bars indicate the standard error of the mean. ** represent significant difference levels of *p* < 0.01. (**C**) Neck blast disease reaction of W3660, WSSM, NTXZ, and YSXSM. Scale bar, 1 cm. (**D**) Statistical analysis of the setting rate of the rice panicles with natural inoculation. The bars indicate the standard error of the mean. ** represent significant difference levels of *p* < 0.01, respectively. WSSM—Wushansimiao. NTXZ—Nantaixiangzhan. YSXSM—Yishenxiangsimiao.

In the context of modern agriculture, it is crucial for novel rice cultivars to exhibit a robust resistance to biotic stresses. To assess the bacterial blight resistance of the newly developed low-glutelin rice variety YSXSM, as well as that of its parental lines, WSSM and NTXZ, experiments were conducted to evaluate their responses to *pathotype IV Xanthomonas oryzae* pv. *oryzae* (*Xoo*) strains (Figure 5A). The results revealed that both YSXSM and its parental lines, WSSM and NTXZ, which possess the *Xa23* allele (Figure 2I), exhibited moderate to high resistance to the *pathotype IV Xoo* strains after artificial inoculation (Figure 5B). In contrast, W3660, which lacks the *Xa23* allele, displayed high susceptibility to the *pathotype IV Xoo* strains. Furthermore, the resistance to blast disease was evaluated through natural inoculation tests (Figure 5C). YSXSM, which carries the *Pi-2* gene (Figure 2H and Figure 5D), displayed a resistance to neck blast that was similar to its parental varieties, indicating its robust resistance against this fungal disease. Conversely, W3660, lacking the *Pi-2* gene, exhibited susceptibility to rice neck blast. In summary, the newly developed low-glutelin rice variety YSXSM, which has the advantage of pyramiding both the *Xa23* and *Pi-2* genes, demonstrated dual resistance to bacterial blight (BB) and blast diseases. This multidimensional resistance capability enhances the adaptability and resilience of YSXSM in the face of common biotic stresses, making it a promising addition to the rice cultivars available for agricultural use.

### 3.4. Quality Improvement of the New Line 

**Figure 6:**
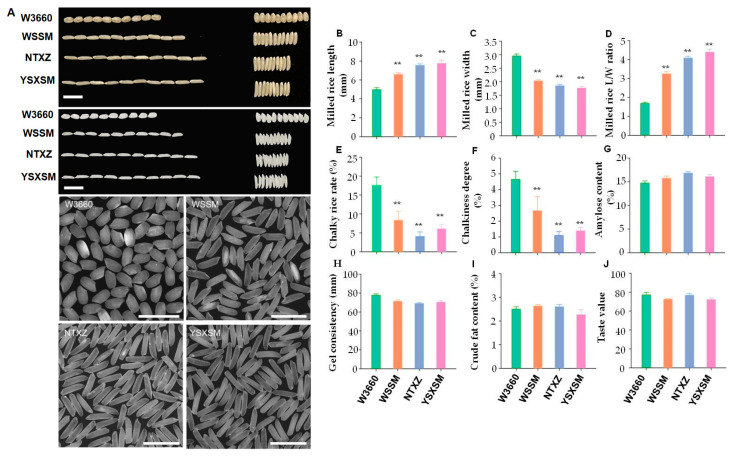
Comparison of grain quality in the new line and parents. (**A**) The performance of the brown grain and milled grain. Scale bars, 1 cm. (**B**) Milled rice length (*n* = 20). (**C**) Milled rice width (*n* = 20). (**D**) Milled rice length/width ratio (*n* = 20). (**E**) Chalky rice rate (*n* = 200). (**F**) Chalkiness degree (*n* = 200). (**G**) Amylose content (*n* = 3). (**H**) Gel consistency (*n* = 3). (**I**) Rice crude fat content (*n* = 3). (**J**) Taste value (*n* = 3). The bars indicate the standard error of the mean. ** represent significant difference levels of *p* < 0.01, respectively. WSSM—Wushansimiao. NTXZ—Nantaixiangzhan. YSXSM—Yishenxiangsimiao.

**Figure 7:**
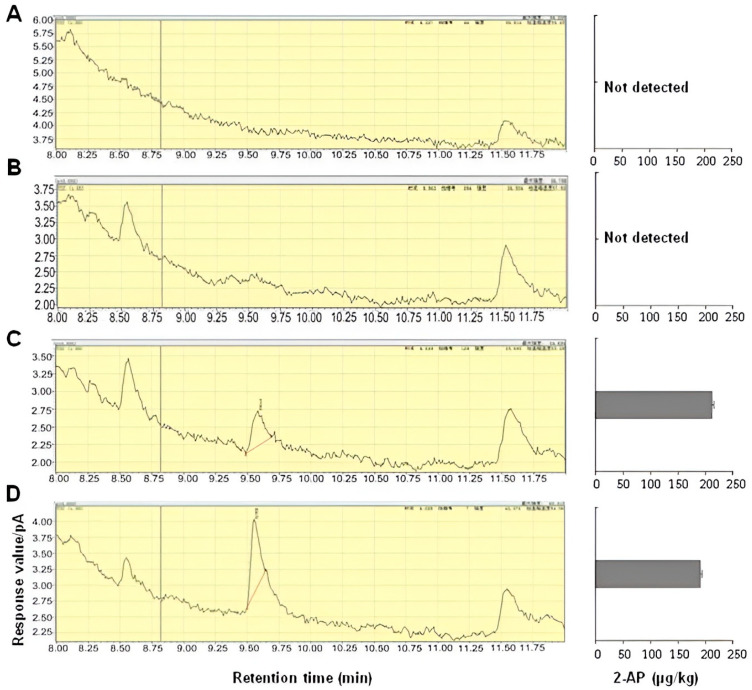
GC-MS TIC profiles and the content of 2-AP in the milled grain. (**A**) W3660, (**B**) WSSM, (**C**) NTXZ, and (**D**) YSXSM. The bar indicates the standard error of the mean.

The parent NTXZ is a superior quality *indica* variety with a good rice appearance and fragrance. In the breeding process, the rice appearance quality (AQ) that was similar to that of the NTXZ was selected based on traditional breeding methods; on the other hand, the trait of fragrance was selected via MAS, and, finally, the new line YSXSM had a greatly improved rice appearance quality compared with W3660 and WSSM (Figure 6A–F). However, there were no significant differences in the amylose content, crude fat content, gel consistency, and taste value between YSXSM and its parents (Figure 6G–J). The determination of 2-acetyl-1-pyrroline (2-AP) in the milled rice using GC-MS analysis showed that the 2-AP content of YSXSM and the parent NTXZ was 189.78 µg·Kg^−1^ and 211.02 µg·Kg^−1^, respectively (Figure 7C,D). In contrast, no 2-AP was detected in W3660 and WSSM (Figure 7A,B). In summary, our data showed that the YSXSM is an elite rice cultivar with a good quality.

### 3.5. RNA-seq Showed the Expression Patterns among W3660, WSSM, NTXZ, and YSXSM, and Partial Gene Expression Levels Revealed That the Particulate Biotic and Lgc-Adjacent Regions Were Selected to Generate YSXSM

**Figure 8:**
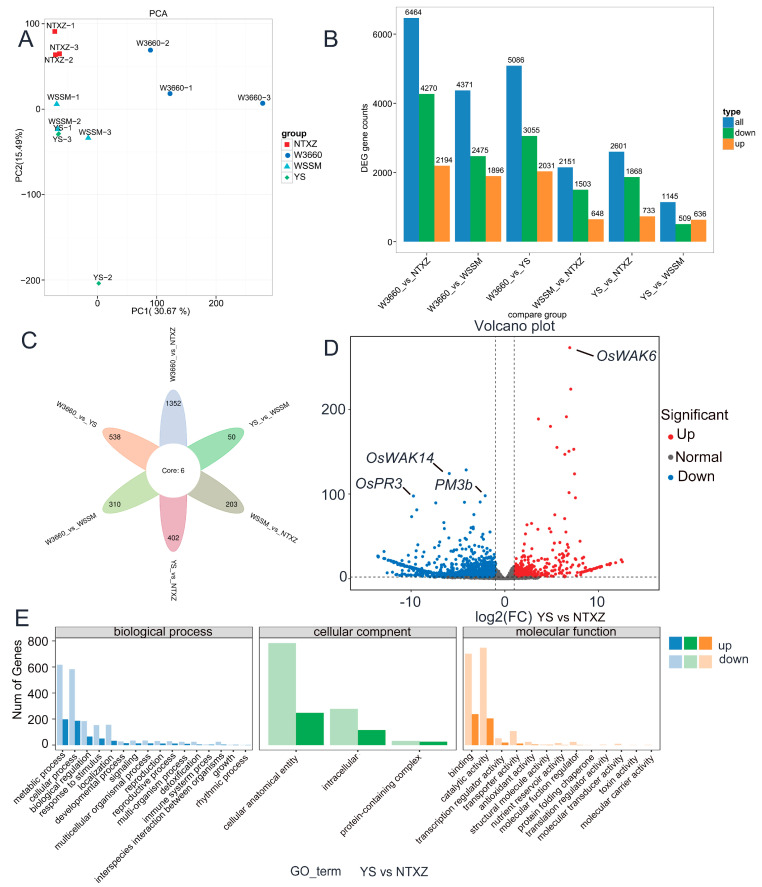
RNA-seq revealed the major expression differences among W3660, WSSM, NTXZ, and YSXSM. (**A**) PCA score plot of two principal components in W3660, WSSM, NTXZ, and YSXSM in the seedling stage. (**B**) Counts of DEGs in the different comparative groups. (**C**) Unique expression of differentially expressed genes appeared in the different groups. (**D**) Volcano map showing differentially expressed genes in comparison to NTXZ and YSXSM. The red dots indicate the up-regulated genes in NTXZ, and the blue dots indicate the down-regulated genes in NTXZ. logFC values over 2 are displayed on the map. (**E**) GO enrichment analysis suggested that YSXSM had improved its biotic resistance compared with NTXZ.

To further validate the molecular differences among W3660, WSSM, NTXZ, and YSXSM, we performed RNA-seq on young seedling-stage leaves of these species. We selected three biological replicates for each sample and conducted RNA-seq and data analysis. The RNA-seq quality control results are included in Supplemental Table S2. After constructing libraries for these samples, we mapped the sequence data onto the Nipponbare reference genome (Supplemental Table S3). Sample correlation and principal component analysis (PCA) indicated that, except for YSXSM-2 (abbreviated as YS-2), the YSXSM groups clustered closely with the WSSM samples, while the NTXZ and W3660 samples formed a separate group (Figure 8A). This result suggests that the RNA expression patterns in YSXSM and WSSM are highly similar, whereas the other two parental lines exhibit distinct differences. In line with the analysis of differentially expressed genes (DEGs) among these four groups, the highest number of DEGs appeared in W3660 and NTXZ, with over 6464 DEGs in this group. In contrast, the lowest difference was observed in YSXSM and WSSM, with nearly 1000 significant differentially expressed genes in this group (Figure 8B). A comprehensive list of all DEGs is provided in the supplemental files (Supplemental Table S4). To illustrate the unique DEGs in each compared group, we generated a Venn diagram (Figure 8C), and we also created a heatmap of all the group samples (Figure S1). These results highlight the genetic background and overall gene expression similarities between WSSM and YSXSM. Interestingly, YS vs. WSSM showed only 50 unique DEGs, while W3660 vs. NTXZ exhibited 1352 unique DEGs (Figure 8C). Considering that NTXZ is the primary parental rice cultivar of YSXSM and shares the most genetic similarity, we created an expression volcano map based on their relative expression levels (Figure 8D). The DEGs and volcano map revealed differential expressions in genes such as *OsWAKs*, *OsPRs*, and *OsPMs* between NTXZ and YSXSM (Supplemental Table S4). Furthermore, we observed GO enrichment between NTXZ and YSXSM, which primarily involved metabolic processes, cellular processes, biological regulation, responses to stimuli, and localization in biological process. In the cellular component, it encompassed cellular anatomical entities, intracellular structures, and protein-containing complexes. For molecular function, the enriched categories included binding and catalytic activity (Figure 8E). Comparative groups of volcano maps and GO enrichment are presented in the supplemental files (Figure S2, Supplemental Table S5). These results indicate that YSXSM possesses higher plant pathogen resistance while maintaining lower glutelin levels. The gene expression patterns related to rice blast and bacterial blight resistance also show slight changes in the young seedling stages.

**Figure 9:**
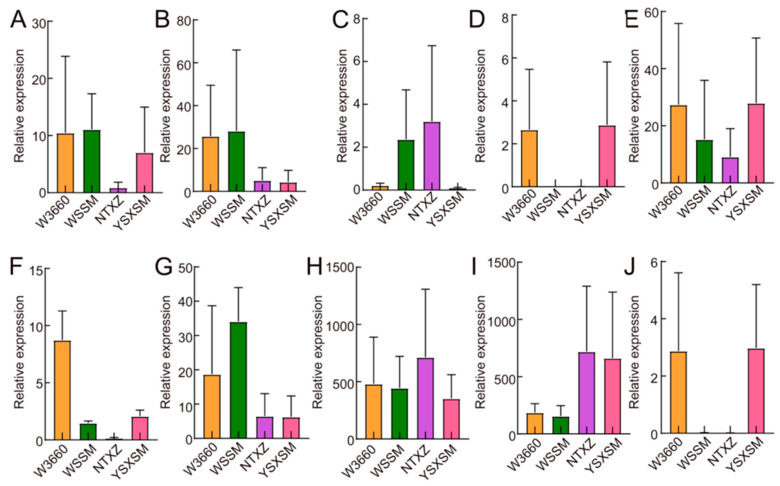
The relative expression levels of certain genes that contributed differently towards W3660, WSSM, NTXZ, and YSXSM. The relative expression of *Pi-2* (**A**), *BADH2* (**B**), *Lgc*-adjacent gene *LOC_Os02g16995* (**C**), *LOC_Os02g16490* (**D**), *LOC_Os02g16040* (**E**), *GIF1* (**F**), *OsRBCS1* (**G**), *OsRCAII* (**H**), *OsRNS4* (**I**), and *Bph14* (**J**). The error bar indicates the FKPM value of the three biological replicates.

Furthermore, we explored some genes related to biotic resistance, location, metabolic processes, and detoxication and generated heatmaps (Figure S3). Upon a deeper examination of the specific gene expression, we identified several interesting phenomena. During the young seedling stage, the *Xa23*, *GluB4*, and *GluB5* genes remained inactive in all four rice cultivars (Supplemental Table S4). Notably, the *Pi-2* genes displayed significant expression differences in W3660 compared with the other three rice cultivars, providing strong support for the effectiveness of our molecular-marker-assisted selection at the RNA level (Figure 9A). Regarding another key selection gene, FGR (also known as BADH2), we observed a variation in its expression in the RNA-seq data (Figure 9B). The low expression of BADH2 in NTXZ and YSXSM may contribute to the grain’s aroma and flavor. Although we did not detect GluB4 and GluB5 expression in the RNA-seq data, we examined nearby genes’ expression in this region. Genes such as *LOC_Os02g16995*, *LOC_Os02g16490*, and *LOC_Os02g16040* exhibited similar expression patterns across all four rice cultivars (Figure 9C–E), suggesting strong artificial selection in this region. Furthermore, we investigated other photosynthetic genes that might influence leaf formation and photosynthesis. *GIF1* displayed a higher expression in W3660 (Figure 9F), while Rubisco (*OsRBCS1*), Rubisco activation enzyme (*OsRCAII*), and the abiotic resistance inducer S-like Ribonuclease exhibited divergent expression levels (Figure 9G–I). These findings suggest that photosynthesis-related genes undergo altered regulation patterns among the four rice cultivars. Finally, we examined the expression of the well-known anti-brown planthopper gene *Bph14*. We found that W3660 and YSXSM might carry the gene resistance allele, leading to a higher expression (Figure 9J). In summary, the RNA-seq data provide a comprehensive and broad understanding of the four rice cultivars’ genetic characteristics and gene expression patterns. 

## 4. Discussion

Unlike common rice, functional rice is a large group of grain products with particular functions or supplementary effects [52]. In addition to having the basic nutrients needed to maintain the normal energy for the human body, it contains certain physiologically active substances or provides higher or lower amounts of special ingredients for body health than common rice in the epidermis, embryo, and endosperm. Functional rice cultivars are capable of balancing the body’s nutrition, preventing disease occurrence, and promoting recovery from diseases after continuous consumption [53]. Low-glutelin-content rice is a special rice cultivar that contains a significantly lower glutelin content than normal cultivars. Moreover, the absorbable protein content is below 4.0%, which is far less than that in normal cultivars, where it is usually over 6.0%. Previous reports have claimed that when ingested, low-glutelin rice effectively reduced the protein intake and serum creatinine in the human body, as determined through clinical trials [15]. On the other hand, low-glutelin rice was found to have a positive effect on the structure and metabolism of the intestinal flora among a healthy population [54]. Therefore, low-glutelin rice has wide application prospects as an economical and beneficial dietary supplement for kidney patient groups and healthy groups.

The glutelin appearing in rice grain is synthesized as a 57 kDa precursor that is subsequently cleaved into a 37–39 kDa acidic subunit and a 22–23 kDa basic subunit in the cytoplasm [55]. This chemical is encoded by multiple genes belonging to four subfamilies, namely GluA, GluB, GluC, and GluD. These synthases are classified based on their amino acid sequence similarity [56]. The analysis of the dominant mutant *lgc-1* revealed that the expression silencing of the GluB gene caused by RNA interference led to a remarkable suppression of GluB protein accumulation, and the result was characterized by lower amounts of mature glutelin (37 to 39 kDa and 22 to 23 kDa in protein body type II) and higher amounts of prolamine (10 kDa, 13 kDa, and 16 kDa in protein body type I) than those in normal varieties [18]. The mutant has been utilized as an essential germplasm resource in the breeding of low-glutelin rice. Recently, a number of new *japonica* rice varieties with low-glutelin content have been generated. However, due to the absence of low-glutelin *indica* rice varieties in tropical and subtropical regions, people have grown accustomed to consuming *indica* rice. Consequently, many kidney patients continue to struggle with accessing commercial low-glutelin *indica* rice. Therefore, there are high demands and requests to breed low-glutelin-content *indica* varieties with a high yield and good agronomic traits to meet the current requirement. In this study, a new low-glutelin *indica* rice variety with the desired comprehensive agronomic performance was developed by improving the low-glutelin *japonica* variety W3660 through gene pyramiding using the MAS method. We confirmed the result via SDS-PAGE electrophoresis and content determination of the grains’ protein. Our data revealed that both the new line YSXSM and its low-glutelin parent W3660 exhibited lower amounts of glutelin (37 to 39 kDa and 22 to 23 kDa) and higher amounts of prolamine (13 kDa), and the content of absorbable protein was significantly below 4%, which consistently meets the characteristics and requirements of low-glutelin-content rice reported thus far.

At present, the goal of modern agriculture is “high and stable yield, good quality and low-pollution” [57]; therefore, it is necessary to develop rice cultivars combining super-high yields, good quality, and multiple resistance. In order to better select the functional rice, a good appearance quality and delicious taste are vital elements to satisfy the consumption needs of patients who use it in their diets. In other words, the development of low-glutelin rice with a high yield potential, multiple resistance, and superior quality could greatly escalate the commercial value. The purpose of this research is to assemble the desired traits, such as a low-glutelin content, high yield, superior quality, and dual resistance, into a single variety by combining MAS and traditional breeding. The genes *Lgc-1* for low glutelin content [17], *Pi-2* for blast resistance [58], *Xa23* for bacterial blight resistance [41], and *fgr* for fragrance [59] were successfully introgressed into an elite *indica* line. Meanwhile, we also focused on a collaborative improvement of other important agronomic traits, and the YSXSM exhibits good comprehensive characteristics, a moderately high yield potential, and a similar growth period to the major cultivars in South China, making it suitable for cultivation in the South China double-cropping *indica* region.

RNA-seq has been a cornerstone technique in plant functional analysis for more than a decade [60]. It has revolutionized our ability to rapidly, affordably, and precisely analyze plant transcriptomes, allowing us to observe dynamic changes at the transcript level. In this study, we conducted RNA-seq on four rice cultivars during the seedling stage, aiming to investigate their genetic distinctions. The PCA results and the counts of differentially expressed genes (DEGs) reaffirmed the genetic divergence among these four cultivars, consistent with our breeding process (Figure 8A,B). Furthermore, we delved into the expression patterns of selected genes among the cultivars. Notably, *Xa23*, *GluB4*, and *GluB5* exhibited no detectable expression in this stage, whereas *Pi-2* and FGR displayed synchronous expression patterns in alignment with their phenotypes (Figure 9A,B). We also examined several genes related to photosynthesis and noted that, in most cases, genes from W3660 exhibited higher expression levels. This phenomenon raised suspicions of background effects. W3660, being a japonica rice cultivar, differs significantly from *indica* rice species. The overall DEG analysis comparing W3660 with other rice species further supported this hypothesis. Of particular interest was the comparison between NTXZ and YSXSM, which showed the fewest unique DEGs, while the genetic similarity between WSSM and YSXSM was the closest. We observed that GO enrichment in these two materials was highly concentrated in the metabolic processes, cellular anatomical entities, and binding functions (Figure 8E). Furthermore, we observed diverse expression patterns in *OsWAKs*, a family of proteins contributing to wall-associated receptor-like kinases [61]. Another key gene related to plant abiotic resistance, *OsPR3*, exhibited enhanced expression in YSXSM, suggesting a potentially higher level of immune reactions in YSXSM compared with NTXZ [62].

These findings provide novel insights into the potential and improvements of YSXSM compared with other rice cultivars. The RNA-seq data illuminate the intricate genetic dynamics in the seedling stage, shedding light on the genetic factors contributing to their distinct characteristics. In recent decades, MAS has been widely used in rice breeding. Target traits can be selected with minimal effects from the environment and allele interactions by genotyping using the tightly linked molecular markers, making MAS an efficient method for realizing polygene polymerization in rice. Although MAS ensures the effective introduction of alleles with major effects, traditional breeding experience is still important to expand the genetic diversity with minor-effect QTLs [63]. As a consequence, integrating MAS with traditional breeding experience is necessary to achieve an efficient and synchronous improvement of comprehensive traits such as yield, quality, and resistance. Recently, CRISPR/Cas technology for multiple genome editing has become increasingly used [64,65,66]. Precisely designed mutants can be derived in a shorter period to achieve similar goals to conventional breeding using CRISPR/Cas in order to achieve targeted mutations on given genes, which could greatly accelerate the research on gene functions and plant breeding. With the arrival of the Industry 4.0 era based on artificial intelligence and big data, more advanced biotechnology will be employed, and designed breeding of complex traits will be easier to implement, which will be conducive to the rapid development of multifunctional rice aggregation. 

## 5. Conclusions

A newly emerged *indica* rice line that pyramided the *Lgc-1*, *Pi-2*, *Xa23*, and *fgr* alleles was developed in this study. The new line retained the desirable features of both parental lines, including a low-glutelin content, dual resistance to blast and BB, and a good quality and fragrance, suggesting that the breeding goal has been achieved. RNA-seq among the four rice cultivars also exhibited comprehensive transcript-level alterations and artificial selection traces. This work provides an available resource for the development of improved functional rice cultivars with multigene aggregation targets for molecular breeding. This cultivar is likely to provide more options for CKD patients in terms of their diet.

## Data Availability

No new data were created or analyzed in this study. Data sharing is not applicable to this article.

## Supplementary Materials

The following supporting information can be downloaded at: https://www.mdpi.com/article/10.3390/plants12213763/s1, Figure S1: The correlation heatmap between samples in this study. Figure S2: The volcano maps of different comparative groups, except Figure 8D. (A) Volcano map of W3660 vs. NTXZ. (B) Volcano map of W3660 vs. WSSM. (C) Volcano map of W3660 vs. YSXSM. (D) Volcano map of WSSM vs. NTXZ. (E) Volcano map of WSSM vs. YSXSM. Figure S3: The heatmaps of different related gene groups among W3660, WSSM, NTXZ, and YSXSM. (A) Heatmap of abitic-related gene groups. (B) Heatmap of detoxification-related gene groups. (C) Heatmap of location-related gene groups. (D) Heatmap of metabolic-process-related gene groups. The number indicates the log10FPKM values of each sample. Supplemental Table S1: Linkage Markers for assist-selection and their primer sequences. Supplemental Table S2: The sequencing data statistics of W3660, WSSM, NTXZ, and YSXSM. Supplemental Table S3: The statistics on data mapping of W3660, WSSM, NTXZ, and YSXSM. Supplemental Table S4: All DEG FPKM values among four rice species. Supplemental Table S5: The DEGs in NTXZ and YSXSM.
