# Supplementary material for: Pyramiding Breeding of Low-Glutelin-Content Indica Rice with Good Quality and Resistance
Source: Plants (Basel). 2023 Nov 3;12(21):3763. doi: 10.3390/plants12213763 (PMC10647759; doi:10.3390/plants12213763)
Supplement: Supplementary file 1 [file plants-12-03763-s001.zip › Supplemental Figure.pptx]

## Slide 1
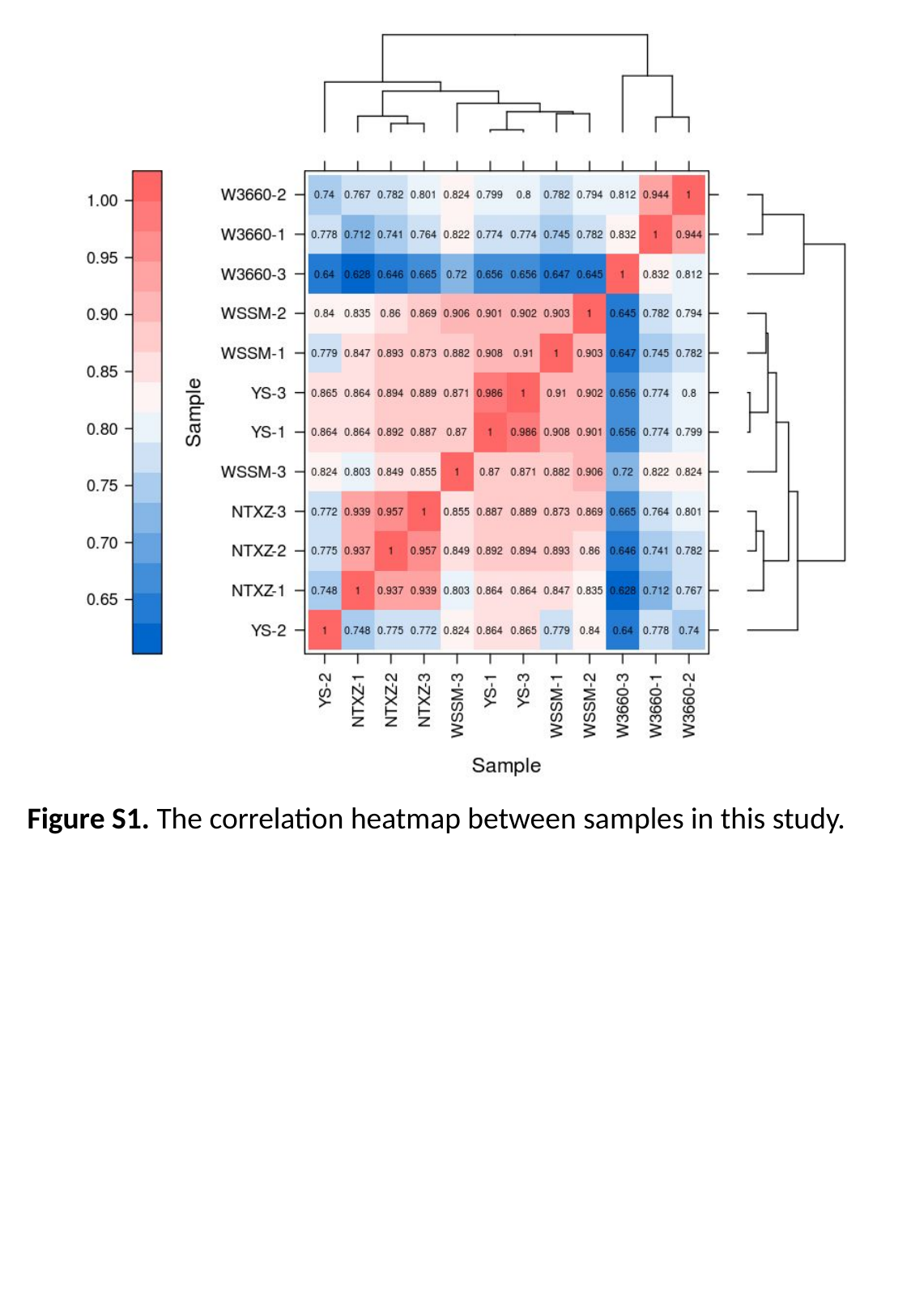

Figure S1. The correlation heatmap between samples in this study.

## Slide 2
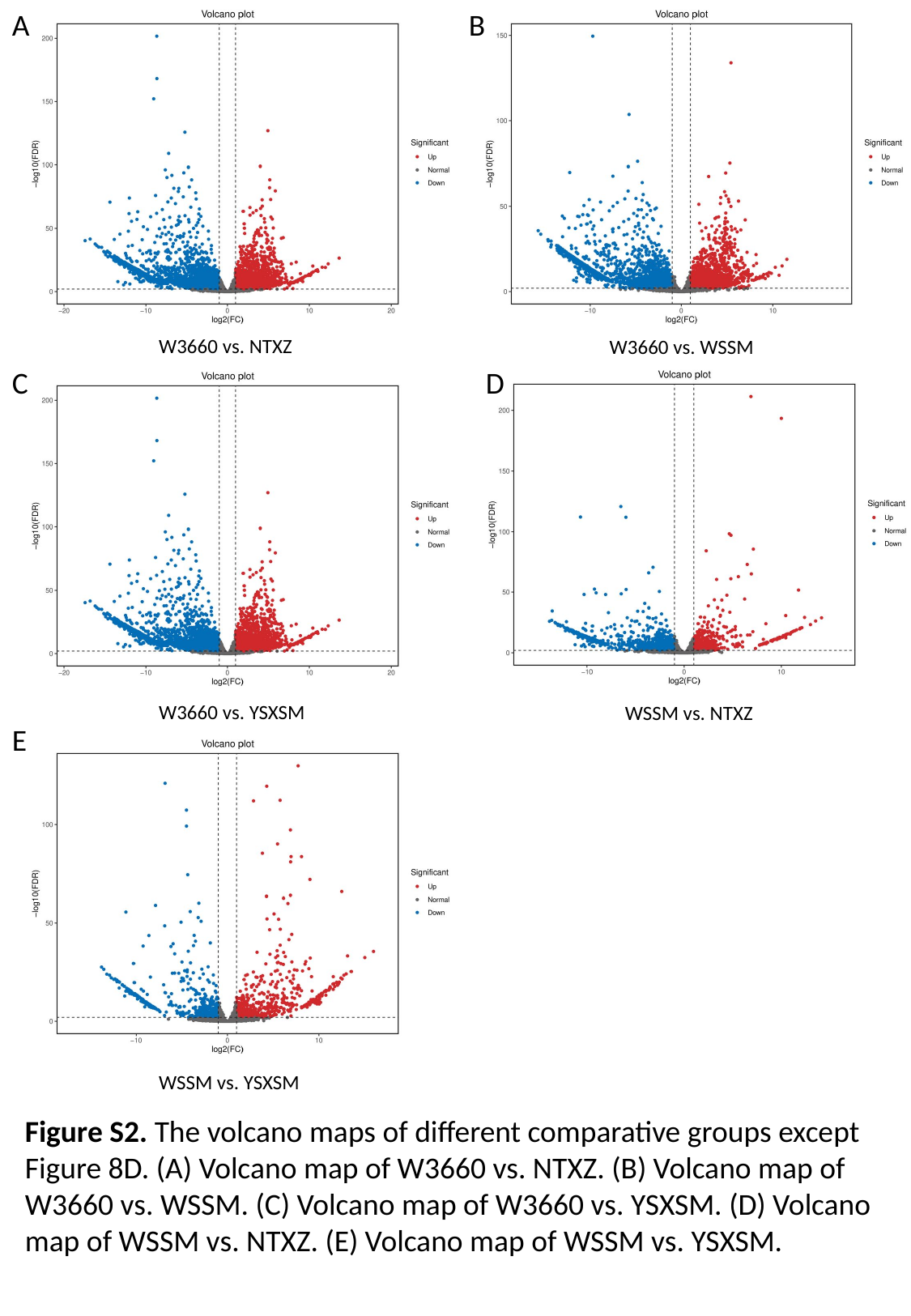

A
B
W3660 vs. NTXZ
W3660 vs. WSSM
C
D
W3660 vs. YSXSM
WSSM vs. NTXZ
E
WSSM vs. YSXSM
Figure S2. The volcano maps of different comparative groups except Figure 8D. (A) Volcano map of W3660 vs. NTXZ. (B) Volcano map of W3660 vs. WSSM. (C) Volcano map of W3660 vs. YSXSM. (D) Volcano map of WSSM vs. NTXZ. (E) Volcano map of WSSM vs. YSXSM.

## Slide 3
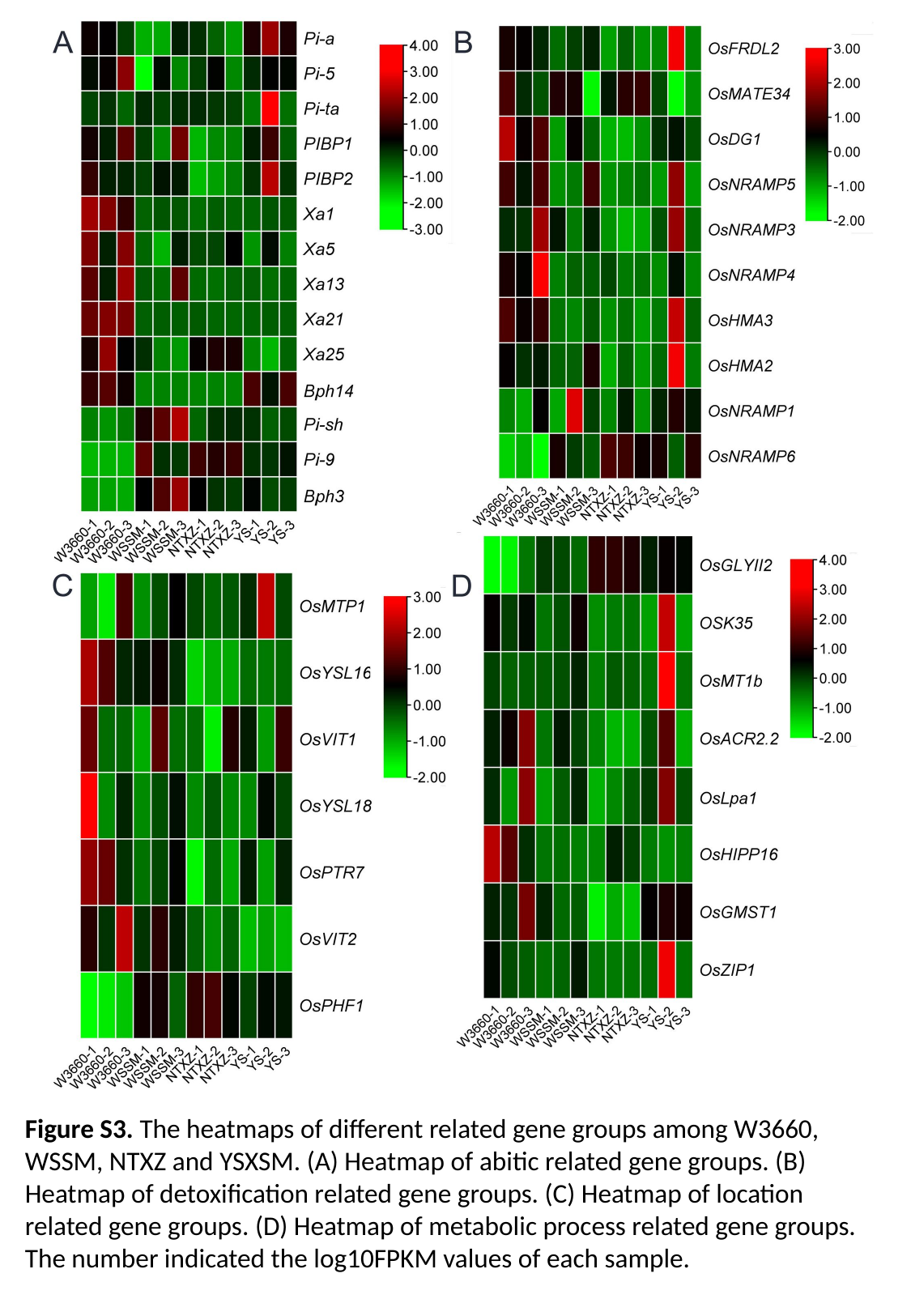

Figure S3. The heatmaps of different related gene groups among W3660, WSSM, NTXZ and YSXSM. (A) Heatmap of abitic related gene groups. (B) Heatmap of detoxification related gene groups. (C) Heatmap of location related gene groups. (D) Heatmap of metabolic process related gene groups. The number indicated the log10FPKM values of each sample.
